# Analytical TCP Model for Millimeter-Wave 5G NR Systems in Dynamic Human Body Blockage Environment

**DOI:** 10.3390/s20143880

**Published:** 2020-07-12

**Authors:** Dmitri Moltchanov, Aleksandr Ometov, Pavel Kustarev, Oleg Evsutin, Jiri Hosek, Yevgeni Koucheryavy

**Affiliations:** 1 Tampere University, 33720 Tampere, Finland; dmitri.moltchanov@tuni.fi (D.M.); aleksandr.ometov@tuni.fi (A.O.); evgeny.kucheryavy@tuni.fi (Y.K.); 2 ITMO University, 197101 St. Petersburg, Russia; kustarev@itmo.ru; 3 National Research University Higher School of Economics, 101000 Moscow, Russia; oevsyutin@hse.ru; 4 Brno University of Technology, 616 00 Brno, Czech Republic

**Keywords:** 5G NR, TCP, mmWave, blockage, analysis

## Abstract

Dynamic blockage of radio propagation paths between the user equipment (UE) and the 5G New Radio (NR) Base Station (BS) induces abrupt rate fluctuations that may lead to sub-optimal performance of the Transmission Control Protocol (TCP) protocol. In this work, we characterize the effects of dynamic human blockage on TCP throughput at the 5G NR air interface. To this aim, we develop an analytical model that expresses the TCP throughput as a function of the round-trip time (RTT), environmental, and radio system parameters. Our results indicate that the blockage affects TCP throughput only when the RTT is comparable to the blocked and non-blocked state durations when the frequency of state changes is high. However, such conditions are not typical for dynamic body blockage environments allowing TCP to benefit from the high bandwidth of 5G NR systems fully.

## 1. Introduction

The 5G New Radio (NR) systems operating in a millimeter-wave band (30–100 GHz) are expected to become an enabling technology for various next-generation mobile systems [1,2]. While their standardization is essentially complete [3], the research focus is shifting towards optimizing the system-level performance.

The use of 5G NR in dense urban scenarios brings unique challenges to system designers. Dynamic blockage of the line-of-sight (LoS) link between the 5G NR base station (BS) and the user equipment (UE) by the moving crowd around the UE leads to increased dynamics in the received signal strength. These fluctuations have been theoretically and experimentally shown to occur at the sub-second timescales [4] and may result in significant changes in the effective data rate provided over the air interface.

The system and user-capacity of mmWave 5G NR systems have been intensely investigated so far.Particularly, the study in [5] reports on maximum theoretical capacity provided to users in the dense deployment of 5G NR BSs. In [6], the authors characterized UE capacity in the presence of multi-connectivity operation. The work presented in [7] derives the NR BS system capacity and UE capacity in the presence of both unicast and multicast sessions. End-to-end simulations of 5G NR systems are reported in [8].

Most of the performance studies so far concentrated on evaluating the system or UE capacity of NR BS deployments. However, it is up to the transport layer protocols to efficiently utilize the resources available. Recent studies have shown that Transmission Control Protocol (TCP) performance over 5G NR systems might be compromised under certain conditions. Utilizing ns-3 system-level simulations, the authors in [9] showed that even though the achieved TCP throughput decreases in the presence of blockage events, no further artifacts are observed, i.e., TCP throughput achieves its theoretical levels in both LoS blocked and non-blocked states. However, the authors in [10] suggested that TCP throughput might degrade dramatically under certain environmental conditions. The work [11] also overviewed this aspect and identified the speed of blockers as the dominant factor behind the performance degradation.

To the best of the authors’ knowledge, no TCP model allowing for an assessment of TCP throughput at the NR air interface has been proposed so far. This paper aims to fill this gap by providing a simple first-order approximation of TCP rate in dynamic blockage conditions under the following assumptions: (i) negligible buffer size compared to the bandwidth-delay product (BDP), (ii) blockage events causing bandwidth drops but no complete outage, and (iii) constant bandwidth available to the TCP session while in the blockage and non-blockage states. To this aim, in this paper, we first propose a simple TCP model in dynamic blockage conditions and then proceed with understanding the effects of radio propagation and environmental parameters on the TCP throughput. The main findings of our work are:

- TCP fails to achieve its theoretical throughput when the round-trip time (RTT) is comparable to blocked and non-blocked state durations. These conditions are seldom observed in the case of dynamic human-body blockage.
- The speed of blockers plays a key role in TCP throughput degradation: higher blocker speed leads to more frequent UE state changes as well as minimizes their durations by eventually reducing the TCP throughput.

This paper is organized as follows. In Section 2, we specify our system model. Next, a TCP model is developed and parametrized in Section 3. The numerical assessment is conducted in Section 4. Conclusions are drawn in the last section.

## 2. System Model

In this paper, we consider a single 5G NR BS having the coverage of *r*, such that UEs do not experience outage conditions when blocked. To calculate *r*, we employ the standardized MCS set [12] and the 3GPP UMi propagation model [13]. The height of the BS is $h_{A}$. The spatial density of humans acting as dynamic blockers is $\lambda_{B}$, and they move according to the random direction mobility (RDM) model [14] with the speed of $v_{B}$ m/s by having the exponentially distributed run length with the mean of $\tau_{B}$ m. Users are modeled as cylinders with a height of $h_{B}$ and a radius of $r_{B}$. The height of the UE is $h_{U}$. Following the measurements of human-body blockage [4], the LoS occlusion by humans is assumed to result in 20 dB of additional signal strength degradation.

We model a selected UE located at the distance of *d*, d<r, from the BS, and provided with the bandwidth of *B*. We are interested in the throughput achieved by a TCP source associated with the selected UE. The mean RTT that includes all of the delays both in a wired network and at the 5G NR interface is assumed to be *D*. Similarly to other studies, for example, [15], we address the congestion control phase, where TCP relies upon the additive increase multiplicative decrease (AI/MD) mechanism. Accordingly, after each RTT, TCP increases the number of transmitted segments by one. Once the capacity is achieved, several segments may be lost, forcing the TCP source to cut down its rate by half. We also assume that the residual packet error rate, caused by imperfect error concealment at the NR physical and data-link layers, is negligible [11].

## 3. TCP in Dynamic Blockage Environments

This section outlines the developed model and related parameters.

### 3.1. Modeling Assumptions

**Figure 1:**
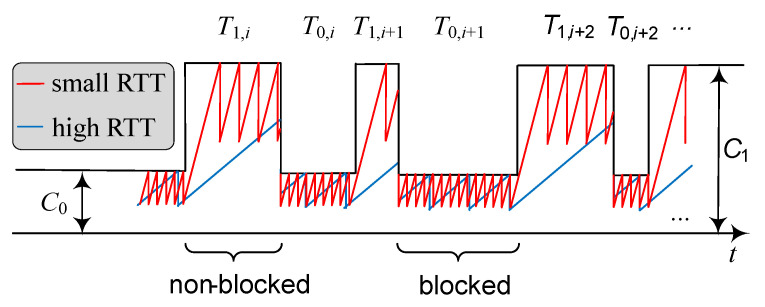
Example Transmission Control Protocol (TCP) throughput in the presence of dynamic blockage.

The intrinsic TCP behavior is determined by the bandwidth-delay product (BDP) of a connection. When the buffer size at NR BS is larger than BDP, TCP is capable of fully exploiting the available bandwidth [16]. However, owing to the extremely large bandwidth and typical RTT in the range of 30–200 ms [17], BDP in 5G NR systems is expected to be large. Furthermore and as shown in [11], operating with large buffer sizes may lead to the bufferbloat effects, negatively affecting latency at the air interface. When the buffer size is negligible compared to the BDP, TCP congestion control leads to “saw-tooth” behavior (see Figure 1) affected by the RTT *D*, where the slope of the rate increment is α=1/D. When blocked and non-blocked durations $T_{0}$ and $T_{1}$ are long enough and/or the RTT is rather small (depending on, e.g., the environment), the TCP source can almost achieve its maximum ergodic rate [18] of 3/4 of the link rate averaged over blocked and non-blocked intervals.

Based on the abovementioned discussion, in what follows, we take the following critical assumptions: (i) negligible buffer size compared to the BDP, (ii) blockage events causing bandwidth drops but no complete outage, and (iii) constant bandwidth available to the TCP session while in the blockage and non-blockage states. We assess the accuracy of the model in Section 4.

The no-outage assumption may narrow the applicability of the model. If an outage actually occurs during a blockage event, the TCP behavior is also affected by timeouts when the outage interval duration is higher than the RTT time. This should almost always lead to an outage for the practical values of RTTs. The reason is that TCP utilizes the exponentially-weighted moving average (EWMA) smoothing algorithm for setting the value of timeout, as discussed in detail in [19], while the outage intervals caused by a blockage event are on order or 300–1000 ms and higher for a practical density of blockers in the environment, as highlighted in [20].

When either $T_{0}$ or $T_{1}$ are small and/or the RTT is large, TCP throughput may deviate from its maximum rate. While the connection properties define the RTT, $T_{0}$ and $T_{1}$ are affected by the environmental conditions, chiefly by the density and speed of blockers [20]. Below, we first characterize the TCP throughput under general $T_{0}$ and $T_{1}$, and then provide estimates of $T_{0}$ and $T_{1}$ as functions of propagation and environmental conditions.

### 3.2. TCP Throughput

Using the geometric argument, see Figure 1, the TCP throughput during the blocked and non-blocked states can be represented as $R_{i}=C_{i}S_{R_{i}}/T_{i}$, i=0,1, where $S_{R_{i}}$ is the mean area under the rate line in blocked (i=0) and non-blocked (i=1) states, $C_{i}$ is the link rate in blocked and non-blocked states, determined by the system parameters [12] and propagation model [13]. Hence, the time-averaged TCP throughput can be defined as

(1) $$\begin{array}{c} R=\frac{C_{0}S_{R_{0}}+C_{1}S_{R_{1}}}{\left(T_{0}+T_{1}\right)}, \end{array}$$

and the task at hand reduces to determining $S_{R_{0}}$ and $S_{R_{1}}$.

**Figure 2:**
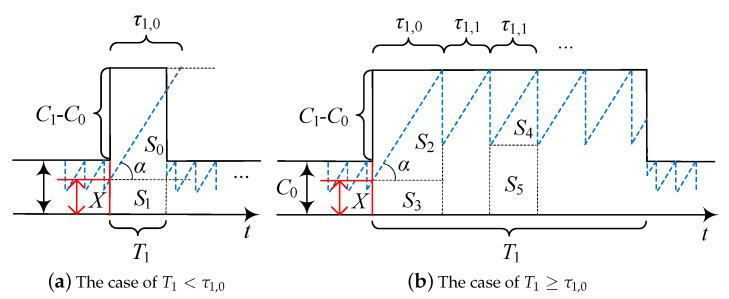
Two possible relations between $T_{1}$ and $\tau_{1,0}$.

Observing Figure 1, one may notice that $S_{R_{0}}$ and $S_{R_{1}}$ can be found by using a similar approach. In what follows, we utilize $S_{R_{1}}$ as an example. We proceed by differentiating between two cases, $T_{1}<\tau_{1,0}$ and $T_{1}\geq \tau_{1,0}$, where $\tau_{1,0}$ is the time for the TCP throughput to reach $C_{1}$, see Figure 2.

Let us first determine $\tau_{1,0}$. As shown in Figure 1,

(2) $$\begin{array}{c} \tan\alpha =\frac{1}{D}=\frac{C_{1}-C_{0}+X}{\tau_{1,0}}, \end{array}$$

where *X* is a random variable (RV). As *X* depends on many system parameters including its previous values, durations and rates in blocked and non-blocked states, RTT, etc., we assume that *X* follows uniform distribution in $\left(0,C_{1}-C_{0}\right)$.

Now, $\tau_{1,0}=D\left(C_{0}-C_{1}+X\right)$, and its mean is given by

(3) $$\begin{array}{c} \tau_{1,0}=\int_{0}^{C_{1}-C_{0}}D\left(C_{1}-C_{0}+x\right)\frac{1}{C_{1}-C_{0}}dx=\frac{D(C_{1}-C_{0})}{2}. \end{array}$$

Consider the case $T_{1}<\tau_{1,0}$ demonstrated in Figure 2a. Here, we have $S_{R_{1}}=S_{0}+S_{1}$. Observe that $S_{0}=\left(T_{1}^{2}\tan\alpha \right)/2$. The area of the rectangle complementing the achieved rate during the non-blocked interval is $S_{1}=T_{1}C_{0}/2$. Using these arguments, substituting tanα=1/D and simplifying, we obtain

(4) $$\begin{array}{c} S_{R_{1}}=\frac{T_{1}^{2}+T_{1}C_{0}D}{2D},\;T_{1}<\tau_{1,0}. \end{array}$$

Consider now the case $T_{1}\geq \tau_{1,0}$, see Figure 2b. The area of interest, $S_{R_{1}}$, comprises of areas $S_{2}$ and $S_{3}$ corresponding to $\tau_{1,0}$, multiple areas $S_{4}$ and $S_{5}$ associated with $\tau_{1,1}$, and a residual area of $S_{4}$ and $S_{5}$ corresponding to the last $\tau_{1,1}$. The areas $S_{2}$ and $S_{3}$ are obtained similarly to (4). The areas $S_{4}$ and $S_{5}$ are fully determined by $C_{1}$, which leads to $S_{4}=C_{1}\tau_{1,1}/4$, $S_{5}=C_{1}\tau_{1,1}/2$. Thus, for $T_{1}\geq \tau_{1,0}$, we establish

(5) $$\begin{array}{c} S_{R_{1}}=\frac{T_{1}^{2}+T_{1}C_{0}D}{2D}+\frac{3\left(T_{1}-\tau_{1,0}\right)C_{1}\tau_{1,1}}{4\tau_{1,1}},\;T_{1}\geq \tau_{1,0}. \end{array}$$

The throughput during the blocked interval is characterized similarly. Omitting the intermediate calculations, we have

(6) $$\begin{array}{c} R_{0}=\left\{\begin{array}{cc} \frac{T_{0}^{2}+T_{0}C_{0}D}{2D}, & T_{0}<\tau_{0,0},\\ \frac{T_{0}^{2}+T_{0}C_{0}D}{2D}+\frac{3\left(T_{0}-\tau_{0,0}\right)C_{1}\tau_{0,1}}{4\tau_{0,1}}, & T_{0}\geq \tau_{0,0}, \end{array}\right. \end{array}$$

where $T_{0}$ is the mean duration of the blocked interval, $C_{0}$ is the maximum rate during this interval, $\tau_{0,0}=DC_{0}/2$ is the time to achieve the rate of $C_{0}$ upon entering the blocked interval estimated similarly to (3), and $\tau_{0,1}$ is time to achieve the rate of $C_{0}$ in the blocked interval.

Combining the results in (4), (5) and (6) we obtain an approximation for TCP throughput. The only unknowns are the means of blocked and non-blocked intervals, $T_{0}$ and $T_{1}$.

### 3.3. Blocked and Non-Blocked Intervals

To completely parametrize our model, one needs to relate $T_{0}$ and $T_{1}$ to the environmental parameters, particularly the speed and spatial density of blockers, $v_{B}$, and $\lambda_{B}$.

**Figure 3:**
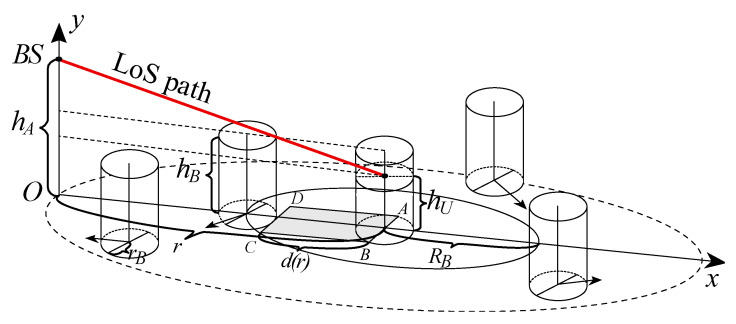
Example dynamic line-of-sight (LoS) blockage phenomenon [5].

Following the recent study in [20], we define a rectangular LoS blockage zone associated with the UE, see Figure 3. The length and width of this zone are given by

(7) $$\begin{array}{c} w=2r_{B},\;l=d\frac{h_{B}-h_{U}}{h_{A}-h_{U}}+r_{B}. \end{array}$$

Recall that the blockers are assumed to move according to the RDM within the coverage area. Using the result in [21], the inter-meeting time of a single blocker with the LoS blockage zone is approximately exponential with the parameter

(8) $$\begin{array}{cc} \gamma_{1} & =v_{B}wl\underset{S_{BS}}{\;\int \int \;}f^{2}\left(s\right)ds=\frac{4r_{B}}{r}\left(d\frac{h_{B}-h_{U}}{h_{A}-h_{U}}+r_{B}\right), \end{array}$$

where $v_{B}$ is the speed of a moving blocker and $f\left(s\right)=1/\pi r^{2}$ is the stationary distribution of the RDM [14].

Since the mean number of blockers within coverage of the 5G NR BS is $\lambda_{B}^{2}\pi r^{2}$ and the inter-meeting time of a single blocker with the LoS blockage zone is exponential, the process of the LoS path blockage is Poisson with the intensity

(9) $$\begin{array}{c} \gamma =4\pi r_{B}r\lambda_{B}\left[d\left(h_{B}-h_{U}\right)/\left(h_{A}-h_{U}\right)+r_{B}\right]. \end{array}$$

Consider now the case where a single blocker occludes the LoS path. Due to the properties of the RDM model, the entry point of a blocker is distributed uniformly over the perimeter of the LoS blockage zone. However, for any reasonable BS to UE distance, we have w<<l, and thus we may disregard the entry and exit points at the short sides of the LoS blockage zone. With these assumptions, the mean path of a blocker in the LoS blockage zone is [22]

(10) $$\begin{array}{c} E\left[L\right]=\frac{2[w^{3}-{\left(w^{2}+l^{2}\right)}^{3/2}]}{3l^{2}}+\frac{w^{2}\ln\left(\frac{l}{w}+\sqrt{1+\frac{l^{2}}{w^{2}}}\right)}{l}. \end{array}$$

As shown in [20], the blocked and non-blocked intervals constitute an alternating renewal process, where the non-blocked time follows an exponential distribution with the mean of $T_{1}=1/\gamma$ [20]. The stochastic properties of the blocked time coincide with the busy period in the M/G/∞ queue having a Poisson arrival process with the intensity of γ and the service times with the pdf of $L/v_{B}$. To offer a closed-form approximation for the TCP throughput, we replace the busy period in the M/G/∞ queue with the one in the M/M/∞ queue having the same mean service time. Utilizing the results of [23], the mean blocked period is

(11) $$\begin{array}{c} T_{0}=\frac{1}{\gamma }\sum_{i=1}^{\infty }\frac{1}{i!}{\left[\frac{\gamma E[L]}{v_{B}}\right]}^{i}=\frac{e^{\gamma E\left[L\right]/v_{B}}-1}{\gamma }, \end{array}$$

which completes the parametrization of the TCP model.

## 4. Numerical Results

In this section, we study the TCP performance in dynamic blockage environments depending on the system parameters, which are provided in Table 1.

### 4.1. Accuracy Assessment

**Figure 4:**
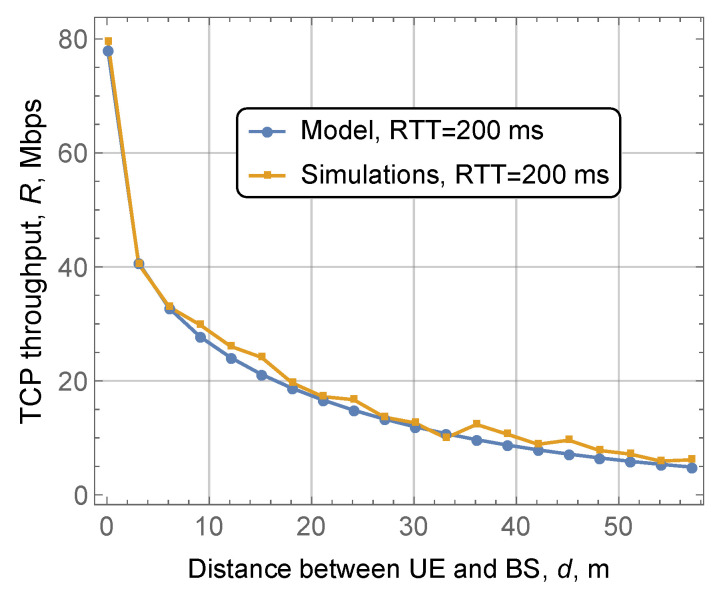
Comparison of model and simulation results.

Further, we assess the accuracy of the developed model. As a simulation tool, we have chosen the mmWave 5G NR cellular network module developed at the NYU Wireless group [24], which has been utilized in [10] for empirical TCP performance evaluation in 5G NR systems. The default ns-3 TCP NewReno implementation with all the associated mechanisms was utilized. We did not disable duplicate acknowledgments in this implementation as the simulated topology presumes a single path towards the destination in a wired part of the network, so that packet reordering does not happen. The rationale behind the choice of this topology is to isolate and investigate the effect of blockage on the 5G NR interface. To this aim, Figure 4 shows a comparison between model and simulation data as a function of the distance between 5G NR BS and UE. The link-layer buffer size has been set to 100 KB to reflect our modeling assumptions, which is much smaller compared to the BDP. As one may observe, the model results match well those obtained using the computer simulations across the whole range of considered distances. Notably, the reported behavior remains true for other input system parameters, including the velocity of UEs, allocated bandwidth, density of blockers in the environment. Thus, in what follows, to demonstrate the effect of these parameters on TCP throughput, we utilize the developed model.

### 4.2. Blocked and Non-Blocked Intervals

**Figure 5:**
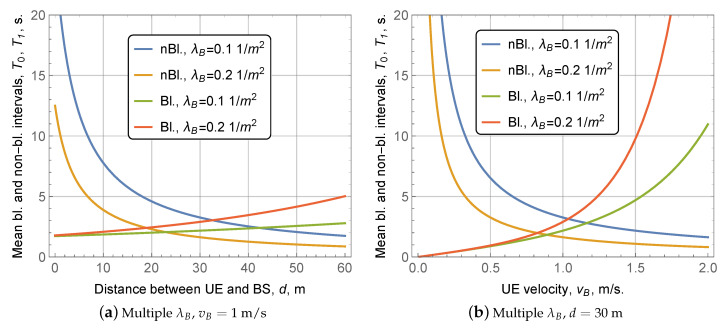
Blocked and non-blocked durations as functions of user equipment (UE)-Base Station (BS) distance and blockers’ density.

**Figure 6:**
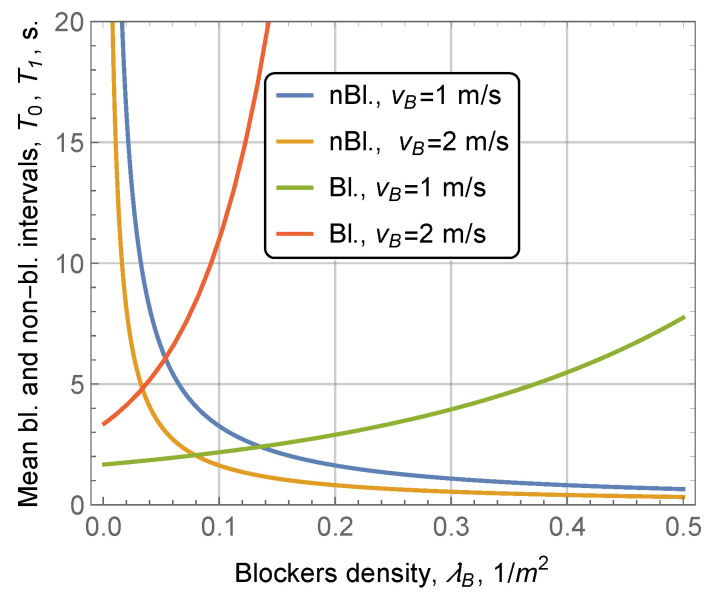
Blocked and non-blocked durations as a function of blockers’ speed.

We proceed by analyzing the impact of the system parameters on the mean blocked and non-blocked durations, see Figure 5 and Figure 6.Observing Figure 5a, one may notice that when the distance between the BS and the UE increases, the mean duration of the non-blocked interval decreases for both of the considered values of $\lambda_{B}$ and $v_{B}$. The reason is that longer distances between the BS and the UE lead to larger LoS blockage zone areas and thus higher temporal intensity of blocker entries. However, the associated increase in the mean blocked interval duration is less drastic. It could be explained by the fact that the effect of higher temporal intensity on the mean blocked interval duration is non-linear as seen from (11). For moderate values of temporal intensity, both intervals are rather short. As it increases further, individual blockers start to form longer blocked intervals. The mean duration of both intervals is always greater than 1 s for the considered distances and values of $\lambda_{B}$ and $v_{B}$. It is significantly higher than the average RTT in the modern Internet, which is often in the range of 30–200 ms [17]. Particularly, it implies one should not expect dramatic TCP throughput degradation, i.e., TCP should achieve its theoretical throughput in both states, for the selected parameters.

Analyzing the dependence of the mean blocked and non-blocked durations on the speed of blockers as shown in Figure 5b, we note that the trend associated with increased speed of blockers is similar to that observed in Figure 5a for the BS to UE distance. The reason is that the growth in $v_{B}$ also affects the temporal intensity of blockers similarly, i.e., increased $v_{B}$ leads to shorter non-blocked intervals and longer blocked intervals. Quantitatively, the effect of $v_{B}$ is more pronounced as compared to the impact of distance. Note that the duration of the blocked interval is short for the small values of $v_{B}$, which implies that TCP may not achieve its theoretical throughput in this state. However, the fraction of time that the UE spends in this state is close to zero, meaning that the TCP throughput degradation is minimal.

The effect of blocker density is demonstrated in Figure 6. Again, the trend caused by $\lambda_{B}$ is similar to that induced by $v_{B}$ and *d*. Comparing the curves corresponding to $v_{B}=1$ m/s and $v_{B}=2$ m/s, we observe that the speed of blockers produces the main impact on the mean blocked and non-blocked intervals.

### 4.3. TCP Throughput

**Figure 7:**
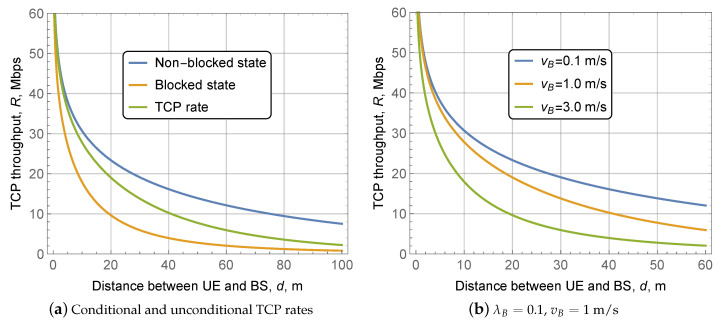
TCP throughput as a function of UE-BS distance.

A comparison of the TCP throughput conditioned on the blocked and non-blocked states as well as the unconditional TCP throughput for $\lambda_{B}=0.1$, $v_{B}=1$ m/s, is shown in Figure 7a. First, we note that the rates in the blocked and non-blocked states differ considerably across the entire range of the considered distances. For smaller distances, when non-blocked intervals dominate, the TCP throughput is close to the one in the non-blocked state. Further, as blocked durations increase, the throughput becomes closer to the one experienced in the blocked state. Such a trend is smooth and has no abrupt changes.

**Figure 8:**
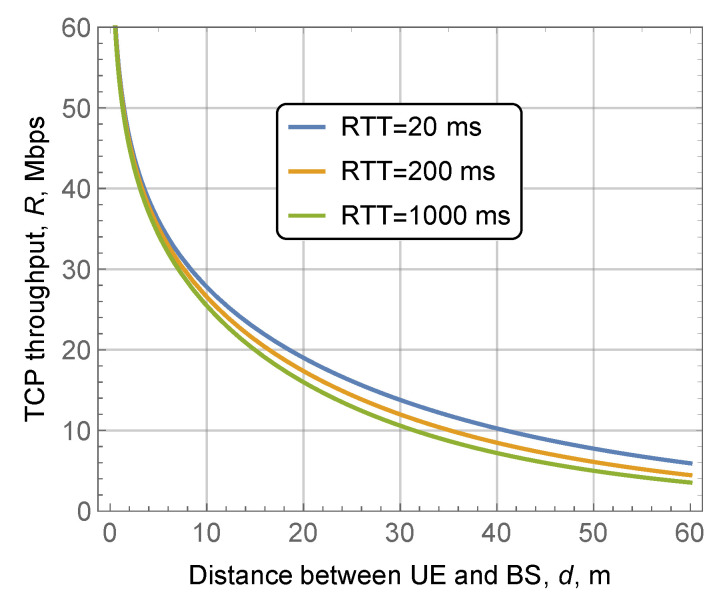
TCP throughput as a function of blocker’s density, $v_{B}=1$.

The effect of blocker speed, $v_{B}$, is assessed in Figure fig:rateTCPb. Since an increase in $v_{B}$ leads to longer blocked periods, higher values of $v_{B}$ result in worse TCP throughput, as it is shown in Figure 5b. Note that the difference is not severe for $v_{B}=0.1$ m/s and $v_{B}=1$ m/s but it increases rapidly for higher blocker speeds. Recalling that the LoS blockage zone grows with the distance between the BS and the UE, the difference between the curves becomes larger for the higher values of *d*. Analyzing Figure 8, one may notice that there is no drastic difference in TCP throughput for the practical values of RTT. The reason is that the RTT in the modern Internet is several times smaller than the mean duration of the blocked and non-blocked intervals, as evident from Figure 5 and Figure 6.

## 5. Conclusions

In this paper, we have developed a simple analytical approximation for TCP throughput achieved at 5G NR air interface in a dynamic human-body blockage environment. The model considers the random movement of blockers and represents the TCP throughput as a function of the environmental and propagation conditions and 5G NR system parameters.

Our numerical results indicate that TCP performance in dynamic human-body blockage conditions is determined by an interplay between the RTT, the frequency of blocked/non-blocked state changes as well as their durations. We have shown that for a wide range of system parameters, TCP achieves its theoretical rate. However, TCP throughput degrades when the RTT is comparable to blocked/non-blocked durations while the frequency of state changes is high. Such conditions are not typical for dynamic human-body blockage environments, allowing TCP to benefit from high bandwidth of the 5G NR air interface.

Among environmental parameters, the blockers’ speed is shown to provide the highest impact on TCP throughput. High blocker speed decreases the durations of blockage and non-blockage intervals and simultaneously increases the frequency of UE state changes. Thus, one may expect significant TCP performance degradation in mobile environments.

## Figures and Tables

**Table 1:** Default system parameters.

Parameter	Value
Carrier frequency	28 GHz
Bandwidth	10 MHz
Transmit power	0.2 W
Propagation model	3GPP UMi [13]
BS/UE antenna gains	14.58 dB, 5.57 dB
LoS blockage loss	20 dB
5G NR BS and UE heights, $h_{A},h_{U}$	4 m, 1.5 m
Blocker height/radius/velocity, $h_{B},r_{B},v_{B}$	1.7 m, 0.3 m, 1 m/s
Spatial blocker density, $\lambda_{B}$	0.1 blockers/m^2^
Round-trip time, *D*	50 ms
Link-layer buffer size	100 KB

## Abbreviations

The following abbreviations are used in this manuscript:

3GPP	3rd Generation Partnership Project
AI/MD	additive increase multiplicative decrease
BDP	Bandwidth-delay product
BS	Base Station
LoS	Line of sight
MCS	Modulation and Coding Scheme
NR	New Radio
RDM	Random walk mobility model
RTT	Round-trip time
RV	Random variable
SIR	Signal-to-interference ratio
TCP	Transmission Control Protocol
UAV	Unmanned aerial vehicle
UE	User equipment
UMi	Urban microcells channel model
